# A novel method for non-destructive determination of hair photo-induced damage based on multispectral imaging technology

**DOI:** 10.1038/srep45544

**Published:** 2017-03-31

**Authors:** Yue Cao, Hao Qu, Can Xiong, Changhong Liu, Lei Zheng

**Affiliations:** 1 School of Instrument Science and Optoelectronic Engineering, Hefei University of Technology, Hefei, 230009, China; 2 School of Biological and Medical Engineering, Hefei University of Technology, Hefei, 230009, China; 3 School of Food Science and Engineering, Hefei University of Technology, Hefei, 230009, China

## Abstract

Extended exposure to sunlight may give rise to chemical and physical damages of human hairs. In this work, we report a novel method for non-destructive quantification of hair photodamage *via* multispectral imaging (MSI) technology. We show that the multispectral reflectance value in near-infrared region has a strong correlation with hair photodamage. More specifically, the hair segments with longer growing time and the same hair root segment after continuous ultraviolet (UV) irradiation displaying more severe photodamage observed *via* scanning electron microscopy (SEM) micrographs showed significantly higher multispectral reflectance value. Besides, the multispectral reflectance value of hair segments with different growing time was precisely reproduced by exposing the same hair root segment to specific durations of UV irradiation, suggesting that MSI can be adequately applied to determine the sunlight exposure time of the hair. The loss of cystine content of photodamaged hairs was identified to be the main factor that physiologically contributed to the morphological changes of hair surface fibers and hence the variation of their multispectral reflectance spectra. Considering the environmental information recording nature of hairs, we believe that MSI for non-destructive evaluation of hair photodamage would prove valuable for assessing sunlight exposure time of a subject in the biomedical fields.

Human hair is natural protein fiber, which is composed of cortex, cuticle and medulla. Cortex is the greatest mass of the hair shaft, which forms 75% to 90% of the hair by weight and contributes to the mechanical properties of hair^1^. The melanin granules, hair pigments, are located inside the cortex (about 3% by weight), whose type, size and quantity are crucial to hair color. The cortex is surrounded by cuticle, a protective layer of overlapping, keratinized scales, which can account for 10% of the hair fiber. As the cuticle protects the cortex against environmental and chemical damage, this region is vulnerable to the extensive damage^2^. Medulla layer is located in the center of the hair fiber, but without active substance.

Along with the growth of hair (varying between 0.7 and 3.6 cm/month with a mean of 1 cm/month generally accepted^3^), there are many factors such as UV irradiation, electric blower, chemical oxidizing and reducing reagent within shampoo, and environmental pollution that can be harmful to human hair. Previous studies^4^ have shown that in the natural environment UV irradiation is the only controllable and the most important factor that leads to hair damage. UV irradiation induced hair damage is called photodamage. UV irradiation weakens the structural integrity of hair fiber, resulting in the degradation of amino acids^5^, oxidation of internal lipid^6^ and melanin granules^7^. Cystine is photo-oxidized into sulfo alanine, which is regarded as an important indicator of the decomposition of hair fiber and in turn affects the mechanical properties of hair fiber^8^.

Considering that hair growth rate is about 1 cm/month, human hair can record and store information of long-term UV irradiation in the natural environment. For example, photodamage of a 5-cm hair close to the scalp represents the amount of UV irradiation in the last five months. So we speculate that the analysis of photodamage of hair segments will help to retrieve historical record of UV irradiation in the natural environment of a subject in the past few months or even years. More specifically, hair photodamage can reflect the exposure to the sunlight, which can regard as an important indicator to evaluate the extent of sunlight exposure for a particular subject, which is crucial to human health since it may have detrimental effects on an individual’s health when there is a deficiency or excess. Long-term sunlight exposure can cause human skin cancer^9^, skin aging^10^, immune suppression^11^ and eye diseases^12^. On the other hand, sunlight is a principal source of vitamin D3^13^, which has a wide range of positive health effects, including strengthening bones^14^ and inhibiting the growth of some cancer^15^. What’s more, sunlight exposure has also been associated with the timing of melatonin synthesis, maintenance of normal circadian rhythms, and reduced risk seasonal affective disorder^16^. Considering the hair photodamage is an important indicator to reflect the individual’s sunlight exposure time, the development of robust and novel methods for hair photodamage detection becomes more and more important for the assessment of the human health.

The way to evaluate hair photodamage includes outward appearance such as hair frizzy, brittle, dry and bifurcation, and chemical indexes such as protein degration, lipidic peroxidation and amino acid degradation^17^. Panda *et al*.^18^ regarded tryptophan as a sensitive marker of hair photodamage, because tryptophan is not stable and easy to be decomposed under UV (296–315 nm). Claerhout S. *et al*.^19^ considered the protein content as an important marker of hair damage, because the internal stable proteins might be turned into denatured proteins under UV irradiation, which were easy to form soluble protein accumulation inside the hair. Nishikawa *et al*.^20^ applied the ^13^C CP/MAS NMR and wide angle X-ray diffraction to study the oxidation of the disulfide bond, which was also an important evaluation basis for hair photodamage. Kuauhara *et al*.^21^ used FT-Raman spectroscopy to study the influence of bleaching on the structure of hair fiber and the disulfide bond in the cortex. Zahn *et al*.^22^ employed amino acid analysis, photometry and polarography for the determination of thiol and disulfide groups in untreated human hair. Richard *et al*.^23^ used the Wilhelmy balance method to measure dynamic contact angle of both conditioner-treated and untreated hairs, and concluded that chemical damaged hairs and untreated hairs exhibited different interaction with water. Ramaparasad *et al*.^24^ used Dia-Stron instrument to measure the hair strength change after bleaching and chemical remediation. M. Richena *et al*.^25^ used SEM, atomic force microscopy and attenuated total reflectance Fourier transform infrared spectroscopy to investigate morphological, ultrastructural and chemical changes after a mercury lamp and small bumps irradiation in the outermost cuticle layer of dark brown hairs. Other factors such as human hair fiber surface wettability^26^, stress-strain measurements^27^ can also be referred to characterize the effect of the UV damage to the hair.

However, the above approaches are almost destructive, complex or time-consuming, and generally require a large amount of hair samples and complicated sample pretreatment. Furthermore, these approaches fail to apply to some special occasions such as the medicolegal investigation and paleontological studies where the hair samples are extremely rare and cannot be damaged. Therefore, it is of greater importance for the development of rapid and non-destructive detection method of photodamage of hairs. Toward this end, we report the application of MSI technology to quantification of hair photodamage and found that the hair segments with longer growing time (i.e. longer exposure time to sunlight) and the same hair root segment after continuous UV irradiation showed significantly higher multispectral reflectance spectra in the near-infrared region. The photodamage of these hair samples was observed and confirmed *via* SEM micrographs. More interestingly, we also found that exposing the same hair root segment to specific durations of UV irradiation could precisely regenerate the multispectral reflectance value of hair segments with different growing time, indicating that MSI could be well applied to determine the sunlight exposure time of the hair. Finally we identified that the morphological changes of hair surface fibers and hence the variation of their multispectral reflectance spectra of photodamaged hairs was physiologically caused by their loss of cystine content.

## Result

### Reflectance spectra of different hair samples

**Figure 1:**
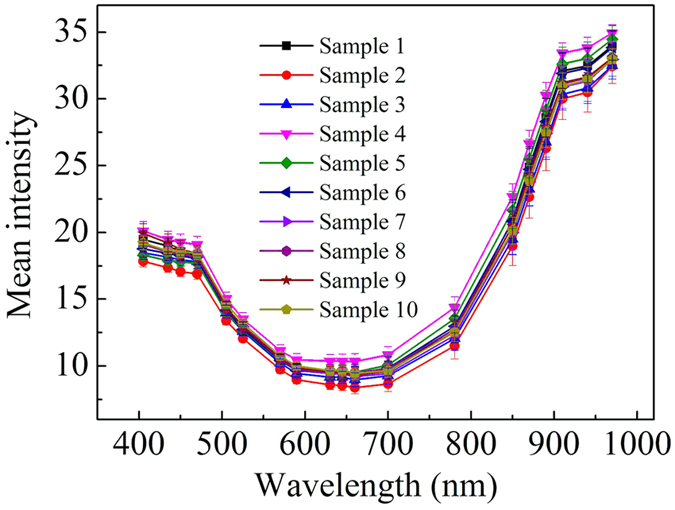
Mean intensity of hairs of ten different samples. Vertical bars represent standard deviations from 50 measurements.

We measured the reflectance spectra of hair samples from different subjects *via* MSI and found that they exhibited significant difference in the near-infrared region, suggesting that MSI could be well applied to human hair samples with satisfactory repeatability. Figure 1 shows the relative reflectance spectra of different hair samples in the range of 405–970 nm. The results indicated that the reflectance spectra of different samples had the same change trend over the entire wavelength range, although it had some difference in value among different samples. In the visible wavelength range (405–700 nm) the difference of reflectance spectra was small, and reflectance value of all samples decreased as wavelength increased. The reason for this small difference could be explained by the slight distinction of color and thickness^28^ of various hair samples. On the contrary, the reflectance spectra in the near-infrared region (780–970 nm) exhibited distinct difference and they all significantly increased with the wavelengths. This large difference was probably caused by the different degrees of photodamage of the hair samples due to various sunlight exposure time of the subjects.

### Reflectance spectra of different hair segments

**Figure 2:**
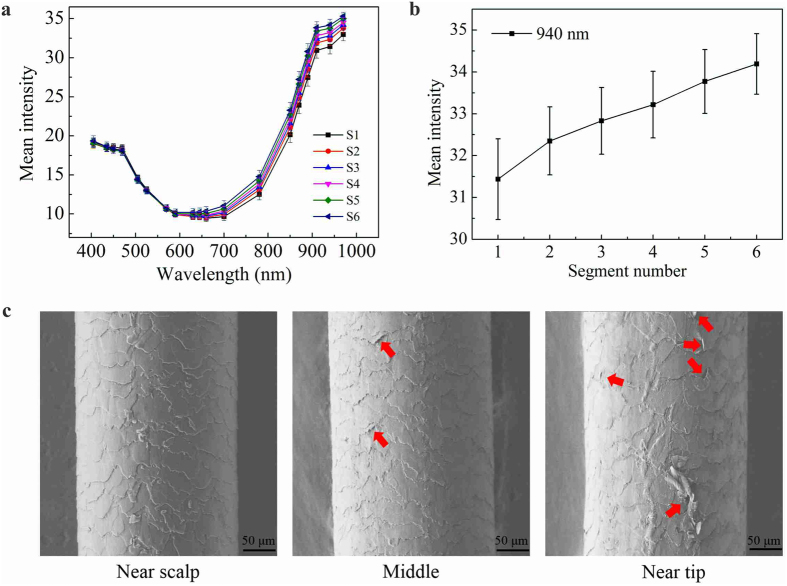
Mean intensity and SEM of different segments in the same sample. Figure 2a is the average reflection spectra of the six successive segments of the hair, Fig. 2b is the change of average reflection spectra at a single wavelength of 940 nm, and Fig. 2c is the SEM images of hair from different region: near scalp, middle and near tip.

We again applied MSI to different segments of the same hair. This was because the average growing speed of human hair was approximate one centimeter per month, and therefore the sunlight exposure time of different parts of the same hair was also different. We found that MSI indeed captured this difference and there was a strong correlation between the reflectance value of MSI and the level of photodamage of the hair, suggesting that MSI was suitable for a rapid and non-destructive monitoring tool for studying hair photodamage. Figure 2 shows that the average reflection spectra from the MSI as well as SEM images of different segments from the same hair sample. Specifically, Fig. 2a shows the average reflection spectra of the six successive segments with an identical length of 5 cm from hair root to end (i.e. S1: 1–5 cm, S2: 6–10 cm, S3: 11–15 cm, S4: 16–20 cm, S5: 21–25 cm and S6: 26–30 cm measured from hair root respectively). Different hair segments had the similar reflection spectra change trend as described before, i.e. a small difference in the visible light region but a sharp distinction in the near-infrared region. Figure 2b displays the average reflection value obtained from the six successive segments of the hair at a single wavelength of 940 nm, at which the average reflection spectra of the different segments showed the maximum difference. The average reflection value at 940 nm had an obvious increasing trend with the segment number.

SEM was also employed to investigate the physical morphology of surface fibers of different hair segments. Figure 2c shows the SEM images of three different segments of the hair: near scalp (S1), middle (S3) and near tip (S6). The cuticles of S1 had intact and tightly overlapping cuticle scales, and the scales had a smooth surface with the typical features described by other authors^29^. Partially detached (as indicated by red arrows), loss of the cuticle edge, and exposure of the cortical cells were observed in S3, indicated by the increase of roughness surrounding cuticle borders, conferring a dirty appearance to the fiber. The cuticle scales of S6 detached seriously, namely, breaking and even falling out from the attached debris. Hair surface morphology observed through SEM indicated that the hair surface damage became gradually serious along the hair from the root to the tip, proportional to the sunlight exposure as the hair grew. Considering that the hair samples were strictly controlled to exclude the effect of other factors (see Methods), the SEM micrographs demonstrated that the increase of the reflectance value along the hair from the root to the tip was mainly due to the increase of photodamage.

### Reflectance spectra of hair before and after UV irradiation

UV irradiation was regarded as the main reason of hair photodamage, causing physical damages including fading color, rough surface, and decrease in mechanical performance, as well as chemical damages such as the change of amino acid, lipids and protein and so on. Physical and chemical damages generally display in the damage and destruction of hair surface fiber.

**Figure 3:**
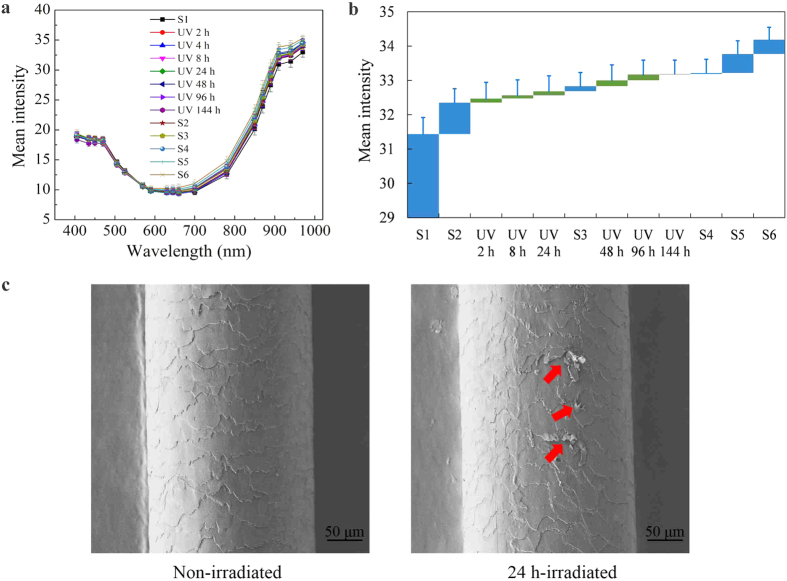
Mean intensity and SEM of a hair before and after UV irradiation. The average reflection spectra of the six successive segments of the hair and the same root segment after UV irradiation shows in Fig. 3a and the change of average reflection spectra at a single wavelength of 940 nm shows in Fig. 3b, and Fig. 3c is the SEM micrographs of the root region of the hair before and after 24 h exposure to UV irradiation

Therefore we studied the effect of UV radiation to the hair photodamage, and found that specific degrees of UV irradiation could precisely reproduce the effect of sunlight in nature to the hair surface. The hair samples were exposed to artificial UV irradiation for different exposure time (see Methods) and the average reflection spectra from the MSI as well as SEM images of the root segment (S1) before and after UV irradiation are exhibited in Fig. 3.

Figure 3a shows the average reflection spectra of the six successive segments of the hair (S1 to S6) and the same root segment (S1) after 2, 4, 8, 24, 48, 96 and 144 h of continuous UV irradiation over the entire wavelength range. Figure 3b displays the average reflection of the hair samples at a single wavelength of 940 nm, which demonstrated that the average reflection of the different segments of the hair could be reproduced by exposing the root segment (S1) to UV irradiation for specific periods of time.

It was notable that the UV irradiation had a strong effect on the microstructures of the hair surface as suggested by SEM micrographs of the same root region of the hair (S1) before and after 24 h exposure to UV irradiation (Fig. 3c). The cuticle scales before UV irradiation appeared in a regular pattern, and each layer was evenly layered. However there was an apparent loss of normalization and evenness of hair surface after 24 h of UV irradiation. Perimeter cracks developed in the cuticle scales, and cuticle scales lifting (indicated by red arrow in Fig. 3c) was clearly observed in the crack region.

This in turn suggests that the hair segmentation analysis can provide a historical record of changes of UV radiation in the natural environment by examining damage to the surface of different segments of hair.

### Cystine content analysis

**Figure 4:**
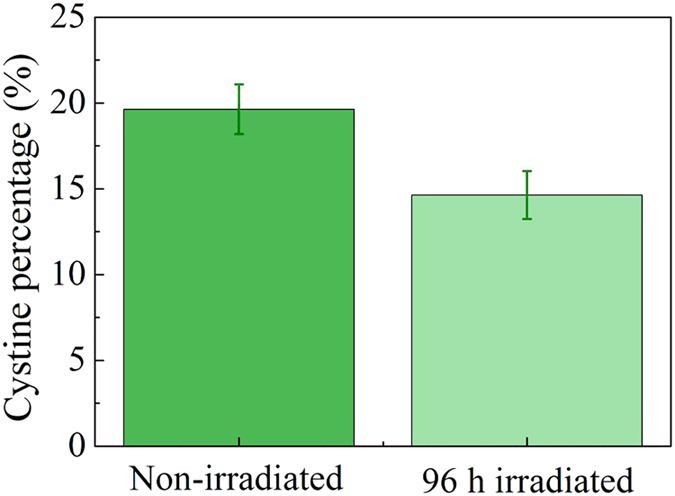
Cystine content obtained from the hairs before and after 96 h of UV irradiation. There was a clear loss of cystine content after 96 h exposure to UV irradiation. Vertical bars represent standard deviations from 3 measurements.

We further investigated the cystine content of hair before and after 96 h of UV irradiation by colorimetry (see Methods) since cystine oxidation was regarded as a main change of chemical composition of hairs caused by photodamage. As shown in Fig. 4, 96 h of UV irradiation indeed led to a significant loss of cystine content of the hair from 19.64% to 14.64%. The loss of cystine content could be one of the main reasons that contributed to serious cuticle damages and low mechanical properties of photodamaged hairs and led to significant difference that appeared in near-infrared region in the multispectral reflectance spectra.

## Discussion

Environmental factors such as UV irradiation, electric blower, environmental pollution, chemical oxidizing and reducing reagent within shampoo are poisonous to human hair. UV irradiation is the most important factor that leads to hair damage in the natural environment, which induces the chemical and physical damages of human hairs. Initial strategies for hair damage evaluation are almost destructive, complex and time-consuming, so it is in urgent need for a rapid and non-destructive tool for evaluating hair damage.

It is particularly noteworthy that there is a strong correlation between the reflectance value of MSI and the level of photodamage of the hair (Figs 2 and 3). As shown in Figs 2a and 3a, the hair segments with longer growing time and the same hair root segment after continuous UV irradiation showed significantly higher multispectral reflectance spectra in the near-infrared region. This result unveils the potential of MSI technology as a rapid and non-destructive method of assaying the extent of damage in hair fibers.

In this study, we report a rapid and non-destructive method to characterize the photodamage of hairs with MSI technique. The morphology of surface fibers of hair samples was quantified by a MSI technology and further characterized with SEM micrographs. We found that the reflectance value of MSI in near-infrared region became significantly higher for the hair samples with more serious photodamage (confirmed by SEM micrographs), namely the hair segments with longer growing time (i.e. longer exposure time to sunlight) and the same hair root segment (S1) after continuous UV irradiation. The multispectral reflectance value of hair segments with different growing time could be precisely reproduced by exposing the same hair root segment (S1) to specific durations of UV irradiation, suggesting that MSI could be applied to determine the sunlight exposure time of the hair. In the end, we identified that the loss of cystine content of photodamaged hairs physiologically contributed to the morphological changes of hair surface fibers and hence the variation of multispectral reflectance spectra. Since mammalian hairs usually have constant growing speeds, long hairs generally record historical environmental information in the past few months or even years. In this way, we believe that the rapid and non-destructive evaluation of photodamages of hairs by MSI would prove valuable for retrieving this information (e.g. assessing sunlight exposure time) of a subject in many fields such as demography, paleontology, forensic medicine and so on.

## Methods

### Hair samples Preparation

In this study, long hair samples (>20 cm) with no history of any chemical treatments such as perm and dyeing and so on were randomly selected from fifty volunteers, and all volunteers gave informed consent before selections. Hair samples were carefully cut with fine scissors as close as possible to the scalp from a posterior vertex position, as studies had demonstrated that these areas had the most consistent linear growth rates^30^. The strands were lined up and cut into six consecutive 5 cm segments (about 5 months growing time) from the root to the tip. The hair segments from the same position of the hairs were stored in the same zip-lock bags and marked as S1 (the segments that were next to scalp), S2 (the adjacent segments), and so on. All hair segments were kept away from light and stored at room temperature until analysis. Immediately prior to the measurements, the hair samples were washed using ultrasonic cleaning with ethanol for 20 min in order to remove oil on the hair surface, and then dried at room temperature. This study was approved by the local Ethics Committee of the Hefei University of Technology, and all experiments were carried out in accordance with approved guidelines of Hefei University of Technology.

### Exposure to artificial UV radiation

Human hairs were irradiated using two 20 W UVB Lamps simulating sunlight radiation, the distance from the lamp to the hair samples was 20 cm and irradiation intensity was about 340 μW/cm^2^ (measured with a radiometer at the same distance from the source as the hair samples). The hair segments next to scalp (S1) were irradiated for 2, 4, 8, 24, 48, 96 and 144 h under UVB lamps. All experiments were conducted at room temperature.

### Multispectral imaging system

**Figure 5:**
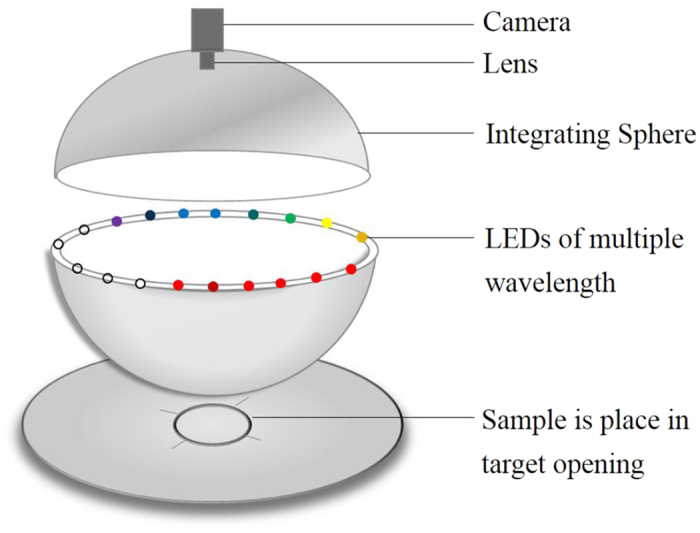
Principal setup of the multispectral imaging system. An integrating sphere with a matte white coating ensures optimal lighting conditions. The LEDs are located at the rim of the sphere. The image acquisition is performed by a CCD camera mounted at the top of the sphere.

Hair samples were analyzed by a MSI system. The MSI analysis was performed using the VideometerLab equipment (Videometer A/S, Hørsholm, Denmark). VideometerLab acquired multispectral images at 19 different wavelengths ranging from the visible region to the lower wavelengths of the near-infrared region at 405, 435, 450, 470, 505, 525, 570, 590, 630, 645, 660, 700, 780, 850, 870, 890, 910, 940 and 970 nm. Figure 5 shows the instrumental setup of the MSI system. The acquisition system recorded the surface reflections with a standard monochrome charge coupled device chip nested in a Point Grey Scorpion camera (Point Grey Research GmbH, Ludwigsburg, Germany). The coating with a matte titanium paint, together with the curvature of the sphere, ensures a uniform reflection of the cast light and thereby a uniform light in the entire sphere^31^. At the rim of the sphere, light emitting diodes (LEDs) are positioned side by side in a pattern which distributes the LEDs belonging to each wavelength uniformly around the entire rim and avoids shadows and specular reflections, an image for each LED of dimensionality 2056 × 2056. The system was first calibrated using a uniform white and dark disc. Then the system was geometrically calibrated with a geometric target to ensure pixel correspondence for all spectral bands^32^. Segmenting images into distinct regions was an important pre-processing step in image analysis. Image segmentation was performed using the VideometerLab software version 2.12.23. To remove the image background, all items except the hair were removed by a Canonical Discriminant Analysis^33^ and segmented using a simple threshold. The image of hair sample without the background was transformed to spectra based on a mean calculation. Thus each image contributed with a single spectrum for the model calibration.

### Scanning electron microscopy

SEM is an intuitive method to observe the hair surface morphology, which has been widely used over many years for claim substantiation of various toiletry treatments on the architecture of the hair’s surface^34^,^35^,^36^. The extent of photodamage of hair surface would be straightforward enough to observe by SEM image. The photodamage of hair surface generally appears as bumps on hair fiber surface, where the cuticles lift, crack and even come off^25^. Scanning electron images of the surface of hair samples were obtained using a high resolution environmental SEM (Hitachi SU8020, SU8020 with excellent low accelerating voltage imaging capability, and directly applicable for nonconductive samples). Every hair strands (3 strands from near scalp, middle, and near tip respectively) with an identical length of 1 cm before and after UV irradiation were fixed on the sample holder stub using a copper adhesive ribbon. Images were obtained under vacuum, using a 1 kV accelerating voltage and 800× magnification. For each sample, at least 10 images were obtained on different areas to ensure the reproducibility of the results.

### Cystine content analysis

A wisp of hairs were irradiated under UVB lamps for 96 h. We then weighed 20 g of untreated-hair and irradiated-hair respectively to prepare for hair hydrolysate. Firstly, 40 mL of 30% hydrochloric acid was used to hydrolyze hair, and the reaction temperature was controlled at 110–115 °C (the boiling temperature of acidolysis solution) during hydrolyzing. After the hair samples were hydrolyzed for 9 h, the liquid phase was filtered at a high temperature and the filtrate was collected. The filtrate was then neutralized with 18% aqueous ammonia to a pH value of 4.8 (cystine isoelectric point), and the solution was stirred constantly so that the reaction temperature was maintained at 40 °C. The precipitate was obtained after 20 h of incubation at room temperature. The precipitate was then completely redissolved with 6% hydrochloric acid, followed by decolorization for 0.5 h under 80 °C using 4% solution quality of activated carbon. The solution was filtered again and the obtained filtrate was hair hydrolyzate. Cystine can reduce Fe^3+^ to Fe^2+^, and Fe^2+^ can react with phenanthroline to form a stable colored ternary complex, which has a maximum absorption value at 510 nm. Therefore the cystine content of the hair hydrolyzate was colorimetrically determined.

## Additional Information

**How to cite this article**: Cao, Y. *et al*. A novel method for non-destructive determination of hair photo-induced damage based on multispectral imaging technology. *Sci. Rep.* **7**, 45544; doi: 10.1038/srep45544 (2017).

**Publisher's note:** Springer Nature remains neutral with regard to jurisdictional claims in published maps and institutional affiliations.
